# Redescriptions of poorly known millipedes (Diplopoda, Chordeumatida) collected by Mr. Hans Sauter in Hokkaido, Japan, with inferences on their type localities

**DOI:** 10.3897/zookeys.1285.186772

**Published:** 2026-07-21

**Authors:** Natsuki Hirakizawa, Ryosuke Kuwahara, Takeo Yamauchi

**Affiliations:** 1 Laboratory of Entomology, Obihiro University of Agriculture and Veterinary Medicine, Obihiro, Hokkaido, Japan Laboratory of Entomology, Obihiro University of Agriculture and Veterinary Medicine Obihiro Japan; 2 Graduate School of Integrated Sciences for Life, Hiroshima University, Kagamiyama, Higashihiroshima, Hiroshima, Japan Graduate School of Integrated Sciences for Life, Hiroshima University Hiroshima Japan

**Keywords:** Conotylidae, *Diplomaragna gracilipes*, Diplomaragnidae, *Japanosoma scabrum*, *Macrochaeteuma sauteri*, Macrochaeteumatidae, taxonomy

## Abstract

Three poorly known species of the millipede order Chordeumatida—*Japanosoma scabrum* Verhoeff, 1914 (Conotylidae), *Macrochaeteuma sauteri* Verhoeff, 1914 (Macrochaeteumatidae), and *Diplomaragna gracilipes* (Verhoeff, 1914) (Diplomaragnidae)—originally described from Hokkaido, Japan, are redescribed based on newly collected specimens. These species were collected in the field for the first time approximately 110 years after their original descriptions. In addition, based on examinations of specimens and a review of historical literature, the type localities of these three species are inferred to be the former Todohokke Village, Hokkaido, Japan.

## Introduction

The order Chordeumatida consists of approximately 50 families, 230 genera, and 1100 species worldwide (Enghoff et al. 2015). In Japan (excluding the southern Kuril Islands), seven families, 10 genera, and approximately 27 species of Chordeumatida are known (Verhoeff 1914, 1929, 1932, 1942; Takakuwa 1949; Miyosi 1958a, 1958b, 1958c; Shear 1987, 1988, 1990; Shear and Tanabe 1994; Shear et al. 1994, 1997; Shear and Tsurusaki 1995; Mikhaljova and Korsós 2015). Of these, three families—Conotylidae, Macrochaeteumatidae, and Diplomaragnidae—have been recorded from mainland Hokkaido, Japan.

Verhoeff (1914) described the following three new species of the order Chordeumatida from Hokkaido, Japan, based on material collected by the German zoologist Mr. Hans Sauter (1871–1943): *Japanosoma scabrum* Verhoeff, 1914 (Conotylidae), *Macrochaeteuma sauteri* Verhoeff, 1914 (Macrochaeteumatidae), and *Diplomaragna gracilipes* (Verhoeff, 1914) (Diplomaragnidae). Of these, *J. scabrum* and *M. sauteri* have not been recorded since their original descriptions. Although *D. gracilipes* has been reported from Korea, Taiwan, and Honshu Island, Japan, these records are highly likely to be based on misidentifications (Shear 1999; Mikhaljova and Lim 2000, 2008; Mikhaljova and Korsós 2015). Further, in the original description, the type locality for all three species was only given as “Japan, Hokkaido” (Verhoeff 1914), leaving their precise collection sites within Hokkaido unknown. Furthermore, the collection dates of these species were not provided. The normal activity period of adult chordeumatidan millipedes is known to extend from late autumn to early spring (Spelda 2015).

In the present study, we redescribe the three poorly known species *J. scabrum*, *M. sauteri*, and *D. gracilipes* based on newly collected specimens, and infer their type localities.

## Material and methods

Specimens were preserved in 70% or 99.5% ethanol and identified under a stereomicroscope (Olympus SZX16) and a microscope (Nikon ECLIPSE Ni). During the study, the gonopods and other selected body parts were dissected and mounted in 70% ethanol as temporary micro-preparations. Some specimens were prepared as slide-mounted specimens in Hoyer’s medium. Specimens were photographed using a single-lens reflex camera (Canon EOS Kiss X10), a digital camera for microscopy (Olympus DP23), and a Scanning Electron Microscope (SEM) (S-3400N, Hitachi High-Tech). Photographs were focus-stacked using Zerene Stacker v. 1.04. For SEM observation, specimens were air-dried and sputter-coated with gold. After examination, the SEM materials were returned to 70% ethanol. Voucher specimens were deposited in the Natural History Museum and Institute, Chiba (CBM-ZU), Japan. Other specimens were stored in the collection of the Laboratory of Entomology, Obihiro University of Agriculture and Veterinary Medicine, Hokkaido, Japan.

Syntypes of *J. scabrum*, *M. sauteri*, and *D. gracilipes* are present in the Zoologische Staatssammlung München (ZSM). We examined images of the syntype specimens of *J. scabrum* and *D. gracilipes* (SNSB 2025a–2025f) available via the Global Biodiversity Information Facility (GBIF).

The distribution map was created using QGIS (v. 3.34). The terminology mostly follows Shear (1987), Spelda (2015), Mikhaljova (2021), and Antić and Mauriès (2022). For *M. sauteri*, we refer to Verhoeff (1914) and Shear (1988).

## Results

### Key to families of Chordeumatida in Hokkaido, Japan

**Table:** 

1	Paranota well developed (Fig. 7A, B), adult body with 32 rings (including collum and telson)	** Diplomaragnidae **
–	Paranota not very well developed (Figs 1A, 1B, 4B, 6A), and adult body with no more than 30 rings	**2**
2	Adult body with 28 rings, body color white, and body length 5.0–7.0 mm	** Macrochaeteumatidae **
–	Adult body with 30 rings, body color pigmented, and body length 7.0–15 mm	** Conotylidae **

In addition to the above families, an unidentified family has also been confirmed, so it is desirable to compare it with gonopod figures.

### Family Conotylidae Cook, 1896

**Subfamily Conotylinae Cook, 1896**

**Genus *Japanosoma* Verhoeff, 1914**

#### Japanosoma scabrum

Taxon classificationAnimaliaChordeumatidaConotylidae

Verhoeff, 1914

E5333110-BE59-5F80-BBD4-C5E1E674A5E3

**Figure 1.**
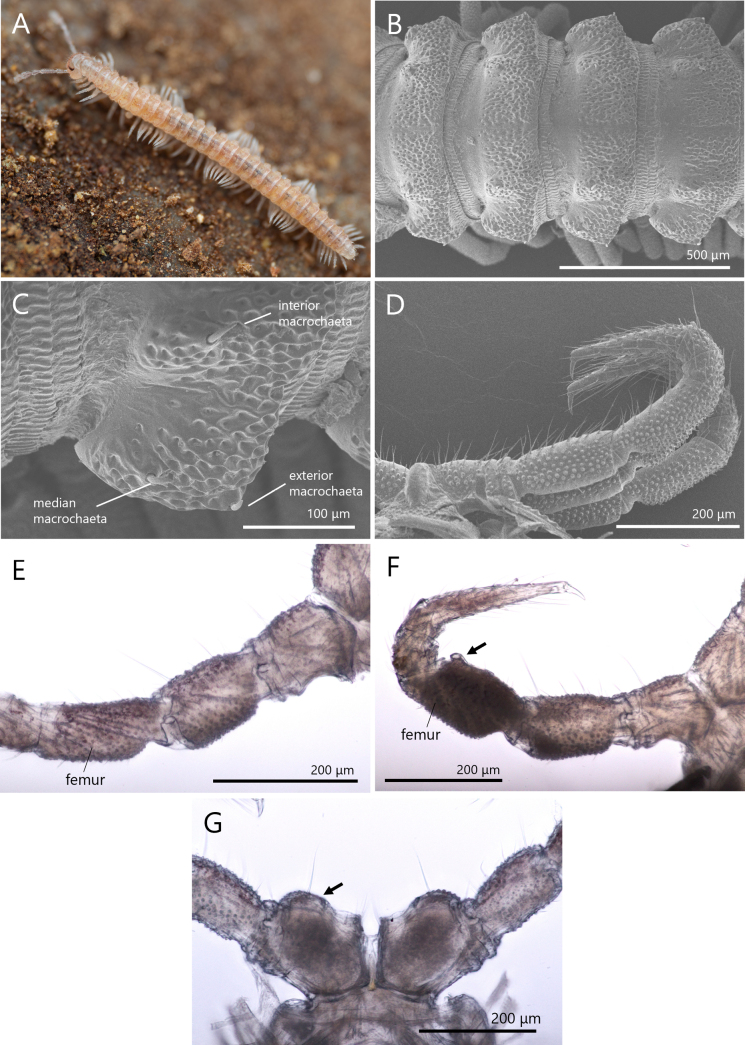
*Japanosoma scabrum*, male adult. **A**. Habitus, live male, dorsal view (CBM-ZU 1350); **B**. Tergites 14–17, dorsal view (CBM-ZU 1349); **C**. Tergite 17, dorsal view (CBM-ZU 1349); **D**. Leg pairs 11–13 (CBM-ZU 1349); **E**. Femur of leg pair 3 (CBM-ZU 1349); **F**. Femur of leg pair 4, showing a cylindrical knob (arrow) (CBM-ZU 1349); **G**. Coxa of leg pair 10, showing a dome-like hump (arrow) (CBM-ZU 1349); **E–G**. Temporarily mounted in 70% ethanol.

**Figure 2.**
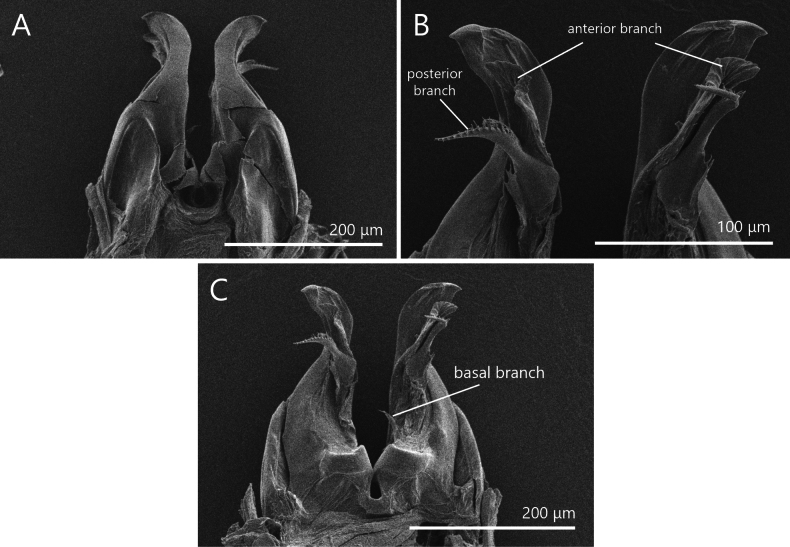
*Japanosoma scabrum*, male adult. **A**. Anterior gonopods, anterior view (CBM-ZU 1349); **B**. Distal part of anterior gonopods, posterior view (CBM-ZU 1349); **C**. Anterior gonopods, posterior view (CBM-ZU 1349).

**Figure 3.**
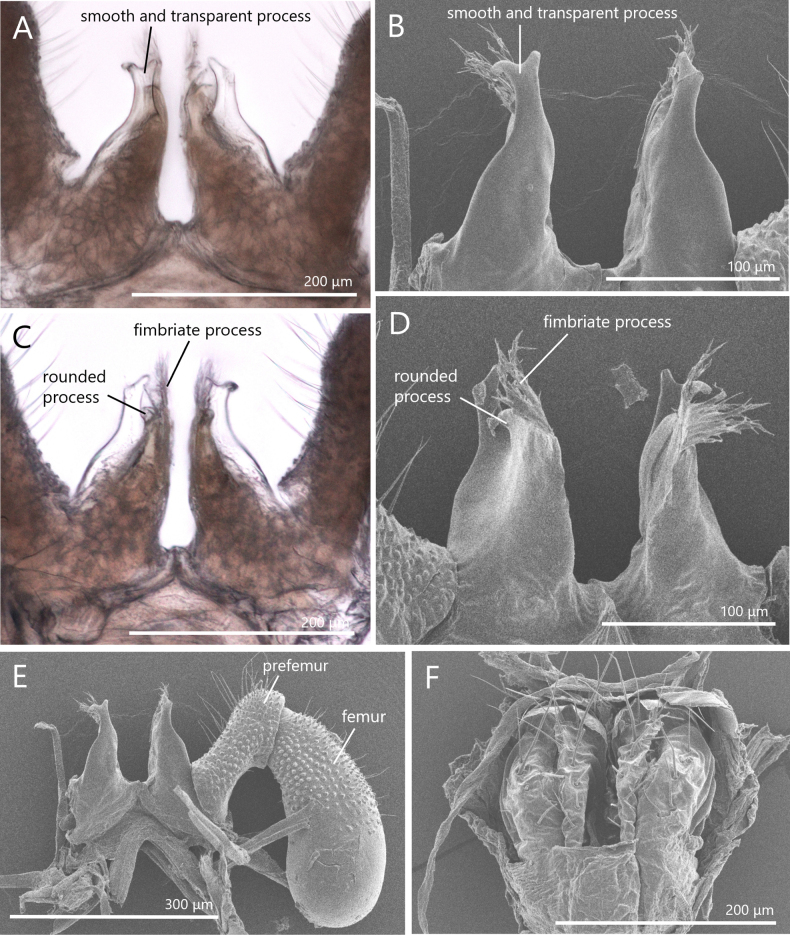
*Japanosoma scabrum*, adult. **A–E**. Male; **F**. Female. **A, B**. Coxa of posterior gonopod, anterior view (CBM-ZU 1349); **C, D**. Coxa of posterior gonopod, posterior view (CBM-ZU 1349); **E**. Posterior gonopods, anterior view (CBM-ZU 1349); **F**. Vulvae, anterior view (CBM-ZU 1348); **A, C**. Temporarily mounted in 70% ethanol.

Figs 1A–G, 2A–C, 3A–F

Japanosoma scabrum Verhoeff 1914: 347, figs 1–4; Takashima 1949: 19 (list); Takakuwa 1954: 122, figs 139, 140; Miyosi 1959: 124, figs 172, 172’; Miyosi 1966: 67; Miyosi 1968: 2; Shear 1987: 22, figs 1–6; Shinohara 1991: 70; Shinohara and Tanabe 1999: 678; Oliveira-Biener et al. 2012: 266, fig. 2; Shinohara et al. 2015: 974.

##### Material examined.

Japan—Hokkaido Prefecture • Hokkaido Island, Hakodate City, Shin-esan-chô; ca. 40 m a.s.l.; 41°49'37"N, 141°08'34"E; 29.III.2025; A. Nomura leg.; 1♂ • same locality; 29.III.2025; A. Nomura leg.; 1♀, CBM-ZU 1348 • same locality; 11.IV.2025; N. Hirakizawa leg.; 1♂, CBM-ZU 1349 • same locality; 11.IV.2025; N. Hirakizawa leg.; 1♀• same locality; 11.IV.2025; R. Kuwahara leg.; 1♂• same locality; 11.IV.2025; N. Hirakizawa and R. Kuwahara leg.; 1♂, CBM-ZU 1350.

##### Description.

**Male**. Body length 7.0–7.7 mm (*n* = 3).

Coloration. Body pale-brown and dull (Fig. 1A). Legs white and antennae whitish purple (Fig. 1A). Ommatidia deep black. Ommatidia number 17–19.

Body with 30 rings (including collum and telson); paranota not very well developed (Fig. 1B). Metatergite very densely covered with deep, minute pits; pits gradually becoming shallower toward the surrounding smooth surface, without sharp boundary (Fig. 1C). Macrochaetae extremely short; three pairs of macrochaetae subequal in length (Fig. 1C).

All legs, except posterior legs, with small warts from coxa to tarsus (Fig. 1D). Leg pairs 1 and 2 slightly reduced in size with tarsal brushes. Seminal openings behind coxa of leg pair 2. Leg pair 3 without modifications (Fig. 1E). Leg pair 4 femur slightly enlarged with cylindrical knob mesially (Fig. 1F). Leg pairs 5–7 without modifications. Leg pair 10 with coxal glands and dome-like humps (Fig. 1G). Leg pair 11 without coxal glands and other modifications.

Anterior gonopods (Fig. 2A–C). In both anterior and posterior view distal parts of angiocoxite curved laterad (Fig. 2A, C). In anterior view, distal part of angiocoxite narrow. In posterior view, bearing three branches; anterior branch with blunt apex (Fig. 2B, C); posterior branch with marginal spiniform projections from midlength to distal end, apex acute (Fig. 2B, C); basal branch small, directed mesially (Fig. 2C).

Posterior gonopods (Fig. 3A–E). In anterior view, coxa with smooth and transparent process, its apex bifid and curved posteriorly (Fig. 3A, B). In posterior view, coxa with rounded process and fimbriate process (Fig. 3C, D). Prefemur and basal half of femur with numerous small, acute warts; femur swollen (Fig. 3E).

**Female**. Body length 8.3–8.4 mm (*n* = 2). Vulvae as in Fig. 3F, external lobe of bursa broader than internal lobe, both lobes with a row of setae. Other somatic characters as in male.

##### Remarks.

The family Conotylidae contains 16 genera and approximately 65 species, distributed in the Nearctic, Japan, and the Russian Far East (Enghoff et al. 2015). In Hokkaido (excluding the southern Kuril Islands), four species (*J. scabrum*, *Yasudatyla hidakaensis* Shear & Tsurusaki, 1995, *Y. shariensis* Shear & Tsurusaki, 1995, and *Y. yasudai* Shear & Tsurusaki, 1995) are known (Verhoeff 1914; Shear and Tsurusaki 1995).

The morphological characteristics of the specimens collected from Shin-esan-chô, Hakodate City, Hokkaido, Japan (formerly Todohokke Village), are consistent with the original description of *J. scabrum* by Verhoeff (1914). *Japanosoma scabrum* (the only species of the genus *Japanosoma*) has not been recorded since its original description; therefore, the present study represents the second record of this species. These specimens were collected under stones and from leaf litter in a forest where a Japanese cedar (*Cryptomeria japonica* (L.f.) D.Don) plantation adjoins a broad-leaved forest, collected in late March and April.

*Japanosoma scabrum* was redescribed by Shear (1987) based on Dr. Verhoeff’s original microscope slide preparations used for the original description (Verhoeff 1914). In addition, extended depth-of-field photographs of the syntype specimen of *J. scabrum* were provided by Oliveira-Biener et al. (2012). Shear (1987: 24, figs 7–9) redescribed the posterior gonopods, stating that “the coxae bear two prominent coxite branches each, the anterior flattened and with posterior fimbriae and the posterior smooth and strongly curved anteriad,”. However, in the present study, smooth and transparent processes were observed on the anterior surface, with apices bifid and curved posteriorly (Fig. 3A, B), while rounded and fimbriate processes were present on the posterior surface (Fig. 3C, D). Although it is difficult to infer the anterior-posterior relationship of the coxal processes of the posterior gonopods from microscope slide preparations (ZSM A20034295 in SNSB 2025a), our SEM observations clarified their positional arrangement. Furthermore, the arrangement of these processes can be confirmed in the extended depth-of-field photographs (Fig. 2D in Oliveira-Biener et al. 2012).

Although not mentioned by Verhoeff (1914) or Shear (1987), a short cylindrical knob was observed on the femur of the male leg pair 4 (Fig. 1F). This knob closely resembles those found on the third and fourth leg pairs of males of the genus *Yasudatyla* Shear & Tsurusaki, 1995 in the family Conotylidae (see Shear and Tsurusaki 1995; figs 1, 2). The characteristics of male leg pairs 3 and 4 in East Asian Conotylidae are summarized in Table 1, and these characteristics are considered useful for genus identification.

**Table 1.** The characteristics of leg pairs 3 and 4 in males of three genera of the family Conotylidae distributed in East Asia (partly based on Shear and Tsurusaki 1995).

	*** Crassotyla ***	*** Japanosoma ***	*** Yasudatyla ***
Leg pair 3	Prefemur with long mesal process; femur enormously inflated	Without modifications	Femur with short cylindrical knob
Leg pair 4	Without modifications	Femur with short cylindrical knob	

### Family Macrochaeteumatidae Verhoeff, 1914

**Genus *Macrochaeteuma* Verhoeff, 1914**

#### Macrochaeteuma sauteri

Taxon classificationAnimaliaChordeumatidaMacrochaeteumatidae

Verhoeff, 1914

C8DEEBF8-D569-5F4E-BF34-CBDDAD5E643E

**Figure 4.**
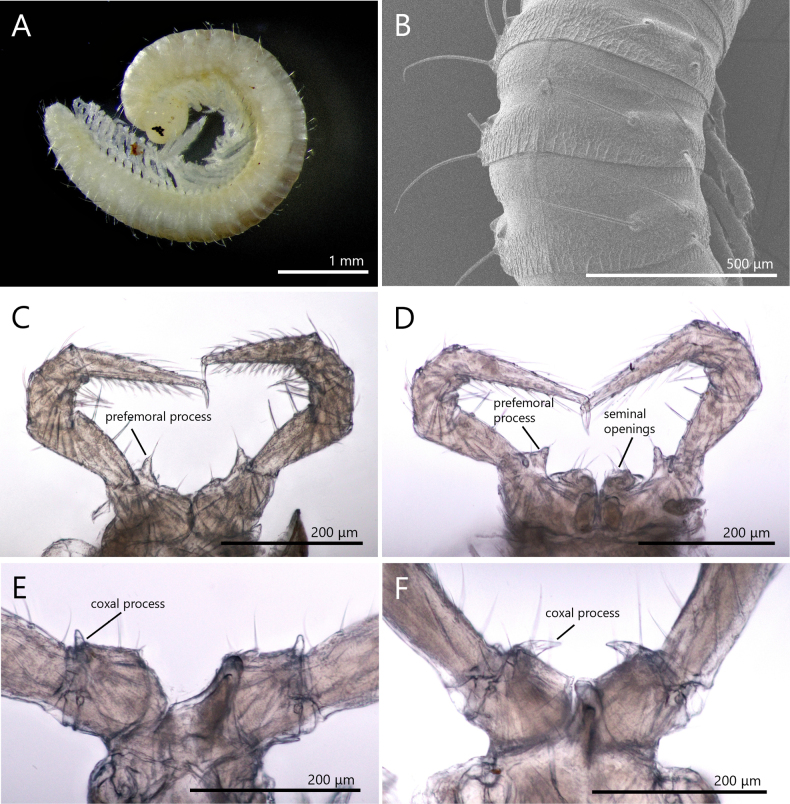
*Macrochaeteuma sauteri*, male adult. **A**. Habitus, lateral view (CBM-ZU 1351); **B**. Tergites 12–14, dorsal view (CBM-ZU 1351); **C**. Leg pair 1 (CBM-ZU 1353); **D**. Leg pair 2 (CBM-ZU 1353); **E**. Coxa of leg pair 4 (CBM-ZU 1354); **F**. Coxa of leg pair 7 (CBM-ZU 1354); **C–F**. Temporarily mounted in 70% ethanol.

**Figure 5.**
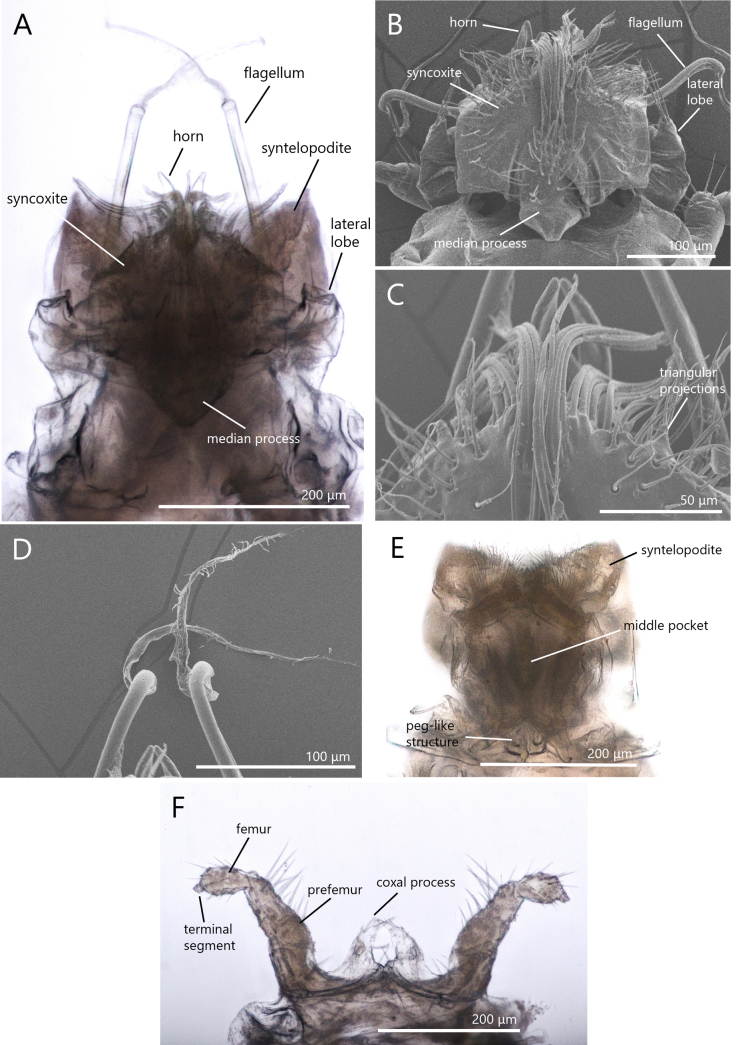
*Macrochaeteuma sauteri*, male adult. **A**. Anterior gonopods, anterior view (CBM-ZU 1353); **B**. Anterior gonopods, anterior view (CBM-ZU 1351); **C**. Tip of anterior gonopods, anterior view (CBM-ZU 1353); **D**. Flagellum of anterior gonopods (CBM-ZU 1353); **E**. Anterior gonopods, posterior view (CBM-ZU 1353); **F**. Posterior gonopods, anterior view (CBM-ZU 1353); **A, E, F**. Temporarily mounted in 70% ethanol.

**Figure 6.**
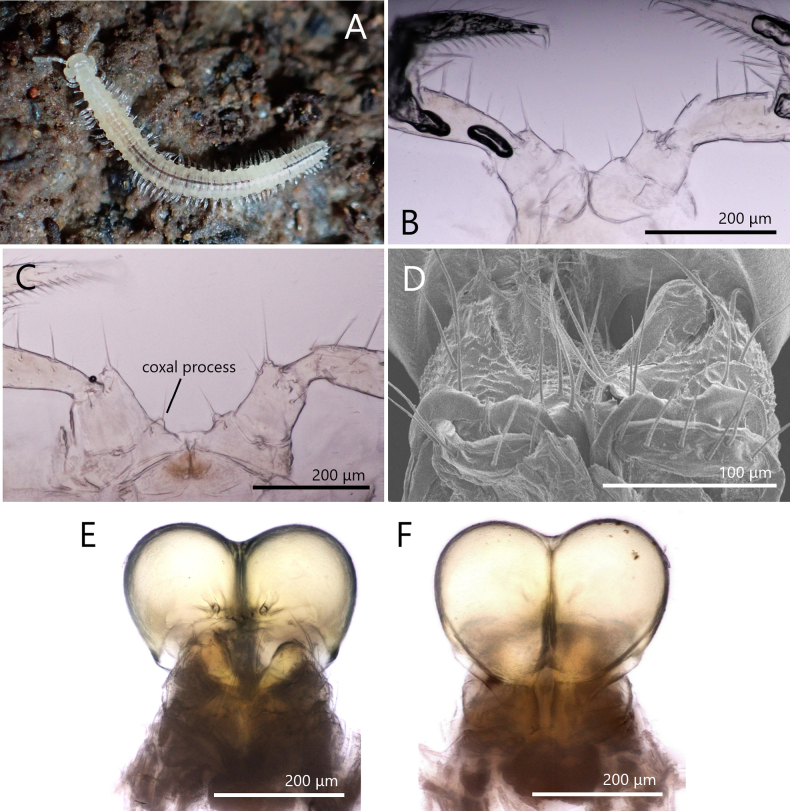
*Macrochaeteuma sauteri*, female adult. **A**. Habitus, live female, dorsal view; **B**. Coxa of leg pair 1 (CBM-ZU 1352); **C**. Coxa of leg pair 2 (CBM-ZU 1352); **D**. Basal part of vulvae, posterior view (CBM-ZU 1352); **E**. Vulvae, posterior view (CBM-ZU 1352); **F**. Vulvae, anterior view (CBM-ZU 1352); **B, C**. Mounted in Hoyer’s medium; **E, F**. Temporarily mounted in 70% ethanol.

Figs 4A–F, 5A–F, 6A–F

Macrochaeteuma sauteri Verhoeff, 1914: 354, figs 5–9; Takashima 1949: 19 (list); Takakuwa 1954: 122, figs 139, 140; Miyosi 1958a: 177; Miyosi 1959: 130, figs 178, 178’, 178’’, 178’’’; Miyosi 1966: 67; Miyosi 1968: 2; Shinohara and Tanabe 1999: 678; Shinohara et al. 2015: 974.

##### Material examined.

Japan — Hokkaido Prefecture • Hokkaido Island, Hakodate City, Shin-esan-chô; 41°49'37"N, 141°08'34"E; ca. 40 m a.s.l.; 29.III.2025; A. Nomura leg.; 1♂1♀, CBM-ZU 1351–1352 • same locality; 29.III.2025; A. Nomura leg.; 1♂ • same locality; 11.IV.2025; N. Hirakizawa leg.; 1♂1♀, CBM-ZU 1353–1354 • same locality; 11.IV.2025; N. Hirakizawa leg.; 1♂.

##### Description.

**Male**. Body length 5.3–6.6 mm (*n* = 4).

Coloration. Body, legs, and antennae completely white (Fig. 4A). Ommatidia deep black (Fig. 4A). Ommatidia number seven.

Body with 28 rings (including collum and telson); paranota not very well developed and metatergite not flat (Fig. 4B). Macrochaetae long, saber-shaped; three pairs of macrochaetae subequal in length (Fig. 4B).

Leg pairs 1 and 2 reduced in size with conical prefemoral process on inner side bearing seta; tarsal brushes present (Fig. 4C, D). Seminal openings behind coxa of leg pair 2 (Fig. 4D). Leg pairs 4 and 7 with conical coxal process (Fig. 4E, F). Leg pairs 3, 5, and 6 without modifications. Leg pairs 10 and 11 with coxal glands, without processes.

Anterior gonopods (Fig. 5A–E). In anterior view, median part of syncoxite with long setae directed upward in upper half and sparse, short setae in lower half; median process directed downward (Fig. 5A). Lateral portions of syncoxite broad, with marginal setae (Fig. 5B). Posterior edge arched medially, with paired triangular projections (Fig. 5C); distal part bearing long setae on both anterior and dorsal surfaces (Fig. 5C). Each side with three branches: lateral lobe (“Seitenlappen” in Verhoeff 1914), horn (“Hörner des Syncoxit” in Verhoeff 1914), and large flagellum (“Pseudoflagella” in Verhoeff 1914) (Fig. 5A, B). Lateral lobe obliquely inclined inward, with 1–3 setae at tip (Fig. 5A, B). Horn slightly curved, apex somewhat acute (Fig. 5A, B). Shaft of flagellum thick and bare; thin distal portion with extremely fine processes (Fig. 5A, D). In posterior view, syntelopodite deformed, with median pocket; base of syntelopodite with two small peg-like structures (Fig. 5E).

Posterior gonopods (Fig. 5F). Prefemur gradually narrowed distally; coxa with process bearing fine setae apically (Fig. 5F). Femur followed by a small terminal segment (Fig. 5F).

**Female**. Body length 6.5–7.5 mm (*n* = 2). Coloration completely white (Fig. 6A). Leg pair 1 with one large seta on inner side of coxa and prefemur, femur with two large and two smaller setae, an enlarged seta on postfemur and tibia (Fig. 6B). Coxa of leg pair 2 with short, seta-bearing process; prefemur with 2–3 setae on inner side (Fig. 6C).

Vulvae (Fig. 6D–F). In posterior view, basal part with a few setae (Fig. 6D, E). Distal part protruding into rounded lobes (Fig. 6E, F) Other somatic characters as in male.

##### Remarks.

The family Macrochaeteumatidae is monospecific, comprising a single genus and a single species, *M. sauteri*, and is known only from Japan (Enghoff et al. 2015).

The specimens collected from Shin-esan-chô, Hakodate City, Hokkaido, Japan (formerly Todohokke Village), are largely consistent with Verhoeff’s (1914) original description of *M. sauteri*. *Macrochaeteuma sauteri* has not been recorded since its original description; thus, the present study constitutes the second record of this species. These specimens were collected from leaf litter in a forest where a Japanese cedar (*C. japonica*) plantation adjoins a broad-leaved forest, adults of *M. sauteri* being collected in April.

In some specimens, the distal part of the lateral lobe of the anterior gonopod appears concave (Fig. 5A), which is likely an artifact of ethanol preservation. This structure is considered to be originally triangular, as in Fig. 5C. Among the four examined individuals, two exhibited a concave shape, whereas the other two retained a triangular shape.

The vulvae (Fig. 6D–F) are consistent with the structure illustrated by Verhoeff (1914), and the rounded lobe may represent a spermatophore.

### Family Diplomaragnidae Attems, 1907

**Genus *Diplomaragna* Attems, 1907**

#### Diplomaragna gracilipes

Taxon classificationAnimaliaChordeumatidaDiplomaragnidae

(Verhoeff, 1914)

199F8274-9837-5795-BB64-3FEFA63096D9

Figs 7A–F, 8A–H

Syntelopodeuma gracilipes Verhoeff 1914: 364, figs 10–14; Verhoeff 1936: 168–170; Verhoeff 1939: 114, 116; Takakuwa and Takashima 1944: 23 (list), 24, 30; Takashima 1949: 19 (list); Takakuwa 1954: 125, figs 142–144; Paik 1958: 362 (list); Wang 1958: 343; Miyosi 1959: 53, 126, 219, figs 173, 173’, 173’’; Miyosi 1968: 2; Ishii 1991: 24.
Syntelopodeuma *gracilis* (sic)—Miyosi 1966: 67.
Diplomaragna gracilipes —Shear 1990: 32, figs 83–87; Lim et al. 1992: 329, 332, 334; Shear 1999: 12, 15; Mikhaljova 2000: 174; Mikhaljova and Lim 2000: 150; Lim 2001: 38 (list), 111, 200 (list), figs 125–129, 249, map 26; Ishii 2004: 395; Mikhaljova and Lim 2008: 58, 60; Mikhaljova et al. 2010: 41, 45; Mikhaljova and Korsós 2015: 570; Nguyen et al. 2016: 45, 46 (list); National Institute of Biological Resources 2019: 597 (list); Kim et al. 2023: 140 (list).

##### Material examined.

Japan — Hokkaido Prefecture • Hokkaido Island, Hakodate City, Egamiyama-chô, 41°50'09"N, 141°07'47"E; ca. 40 m a.s.l.; 6.X.2023; N. Hirakizawa leg.; 2♂1♀, CBM-ZU 1355–1357 • same locality; 6.X.2023; R. Kuwahara leg.; 1♂, CBM-ZU 1358.

##### Description.

**Male**. Body length 14.3 mm (*n* = 1).

**Figure 7.**
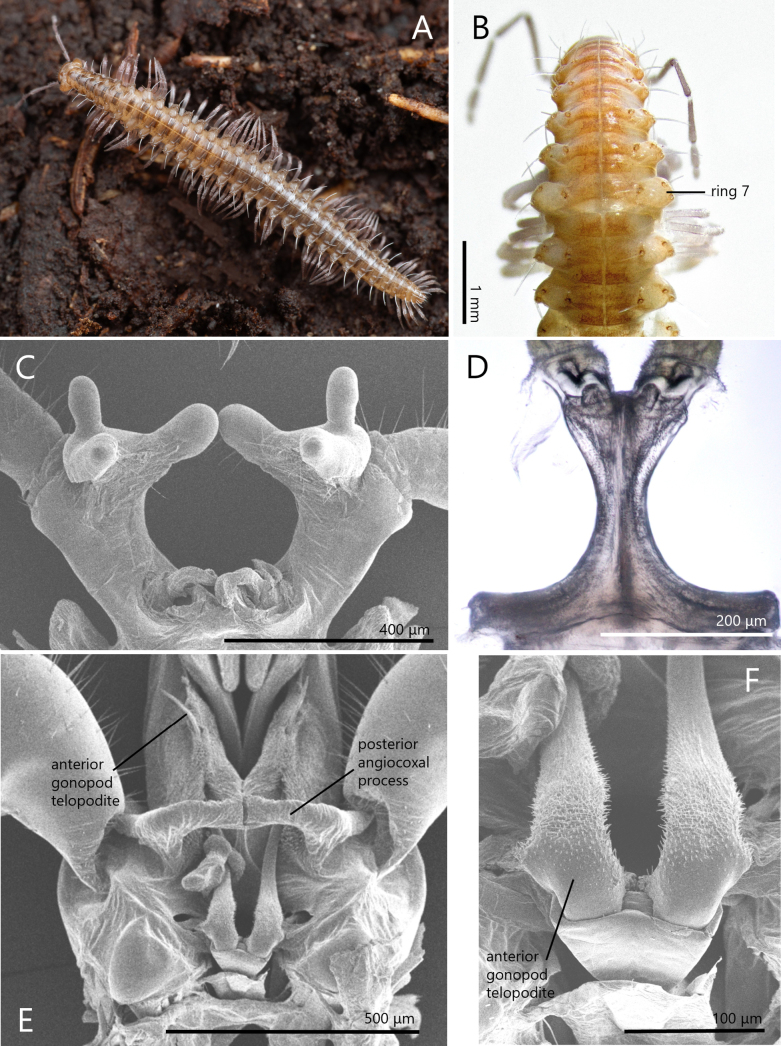
*Diplomaragna gracilipes*, male adult. **A**. Habitus, live male, dorsal view (CBM-ZU 1358); **B**. Tergite, dorsal view (CBM-ZU 1356); **C**. Coxa of leg pair 11 (CBM-ZU 1356); **D**. Coxosternum (CBM-ZU 1357); **E**. Gonopods, posterior view (CBM-ZU 1356); **F**. Base of anterior gonopod telopodite, posterior view (CBM-ZU 1356); **D**. Temporarily mounted in 70% ethanol.

Coloration. Body clay-yellow (Fig. 7A, B). Legs and antennae light purple (Fig. 7A, B). Ommatidia black. Ommatidia number 27–29.

Body with 32 rings (including collum and telson). Paranota well developed, especially on ring 7 (Fig. 7A, B). Metatergite mostly smooth. Macrochaetae long; exterior macrochaetae longest, directed obliquely posteriorly; median and interior macrochaetae subequal in length (Fig. 7A, B).

Legs long and slender (Fig. 7A). Leg pairs 1 and 2 reduced in size with tarsal brushes. Seminal openings behind coxa of leg pair 2. Leg pairs 10 and 11 with coxal glands. Coxae 10 without modifications. Coxae 11 with three finger-like projections (Fig. 7C).

**Figure 8.**
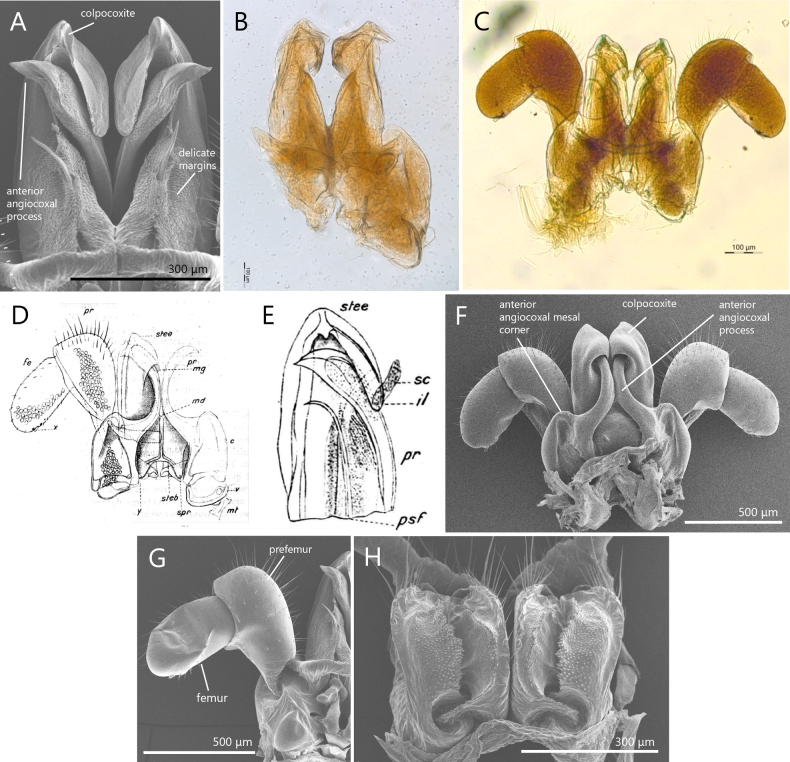
*Diplomaragna gracilipes*, adult. **A–G**. Male; **H**. Female. **A**. Distal half of gonopods, posterior view (CBM-ZU 1356); **B**. Gonopods, syntype (ZSM A20032259), posterior view (from SNSB, 2025d); **C**. Gonopods, syntype (ZSM A20034297) (from SNSB, 2025f); **D**. Gonopods, anterior view (from Fig. 10 of Verhoeff 1914); **E**. Distal half of gonopods, posterior view (from Fig. 13a of Verhoeff 1914); **F**. Gonopods, anterior view (CBM-ZU 1356); **G**. Femur and prefemur of posterior gonopod telopodite, posterior view (CBM-ZU 1356); **H**. Vulva, anterior view (CBM-ZU 1355).

Anterior gonopods (Fig. 7D–F): coxosternum bottleneck-shaped medially, with widened basal region (Fig. 7D). Anterior gonopod telopodite slender, single-membered (Fig. 7E), gradually tapering distally, and distal part sharply pointed; basal portion slightly thickened, covered with spinules (Figs 7E, 7F, 8A).

Posterior gonopods in anterior view (Fig. 8F): anterior angiocoxal processes passing mesially between colpocoxites, through notches on each side. Anterior angiocoxal process slightly robust, distally acuminate and undivided (Fig. 8A). Anterior angiocoxal mesal corner rounded (Fig. 8F).

Colpocoxites basally fused, undivided (Fig. 8F). Colpocoxite tip slightly curved posteriorly (Fig. 8A). In posterior view, colpocoxites distally sheathing distal part of anterior angiocoxal process (Fig. 8A); proximal delicate margins lying close along anterior gonopod telopodite (Fig. 8A). Posterior angiocoxal process simple and narrow, meeting in midline (Fig. 7E). Posterior gonopod telopodite two-segmented; prefemur club-shaped and femur ovoid (Fig. 8G).

**Female**. Body length 13.5 mm (*n* = 1). Legs more slender than in male; paranota of ring 7 less developed than in male. Vulvae (Fig. 8H): operculum distally bilobed; inner valve significantly narrower than outer one, widest at caudal and cranial end, narrowest at place where both vulvae meet; both valves separate by a distinct depression and each showing a row of setae at outer margin, surface of valves between both rows of setae with warty to spiculiform outgrowths, which cover most of surface of outer valve, forming a rectangular field, while restricted on a ridge at inner valve; inner caudally with an outward-facing ridge that fits into a cavity of outer valve. Other somatic characters as in male.

##### Remarks.

The family Diplomaragnidae includes 14 genera and at least 95 species, distributed across the Asian part of Russia, Kazakhstan, Mongolia, Japan, Korea, and Taiwan (Mikhaljova 2021). In Hokkaido (excluding the southern Kuril Islands), four species—*D. gracilipes*, *D. hokkaidensis* (Verhoeff, 1939), *D. tsurusakii* Shear, 1990, and *Tokyosoma takakuwai* Verhoeff, 1929—are known (Verhoeff 1914, 1939; Shear 1990; Nishikawa and Murakami 1991).

The specimens collected from Egamiyama-chô, Hakodate City, Hokkaido, Japan (formerly Todohokke Village), are largely consistent with Verhoeff’s (1914) original description of *D. gracilipes*. These specimens were collected from leaf litter in a Japanese cedar (*C. japonica*) plantation; adults of *D. gracilipes* were collected in October.

*Diplomaragna gracilipes* was redescribed by Shear (1990) based on Dr. Verhoeff’s original microscope slide preparations. Shear (1990) stated that the type material consisted of four microscope slides, but did not provide specimen numbers. Comparison with images available via GBIF (SNSB collection) indicates that these four slides correspond to ZSM A20032257–A20032260 (SNSB 2025b–2025e), detailed as follows: (1) head of a male and leg pairs 1–4 (ZSM A20032260); (2) gonopods, leg pairs 5 and 6, and fragments of tergites (ZSM A20032259); (3) leg pairs 7–12 and fragments of tergites (ZSM A20032258); and (4) head of a female, leg pairs 1–6, and fragments of tergites (ZSM A20032257). However, the SNSB collections also include a fifth microscope slide that was not mentioned by Shear (1990). This slide is considered to represent the specimen illustrated by Verhoeff (1914), as he illustrated the gonopods not separated into parts, except for the coxosternum (Fig. 8D, E).

Shear (1990) illustrated the tips of the posterior gonopod colpocoxites as unciform (largely curved posteriorly), probably based on Fig. 8B (ZSM A20032259 in SNSB 2025d). In contrast, SEM observations of specimens collected in the present study reveal that the colpocoxites are not unciform; rather, their tips are only slightly curved posteriorly (Fig. 8A), consistent with both Verhoeff's (1914) original description and the image of the syntype specimen (Fig. 8C, E; ZSM A20034297 in SNSB 2025f). Since the slide of ZSM A20034297 was not included among the four slides listed by Shear (1990), it is likely that he did not examine the specimen during his redescription.

In summary, these findings suggest that the colpocoxite tips in the slide specimen redescribed by Shear (1990) may have become unciform due to artificial manipulation, such as compression during slide preparation. In *D. gracilipes*, the colpocoxite tips are therefore more likely to be only slightly curved posteriorly, as illustrated by Verhoeff (1914) (Fig. 8E) and in the present study (Fig. 8A).

Although *D. gracilipes* has been recorded from Korea (Takakuwa and Takashima 1944; Takakuwa 1954; Paik 1958; Lim et al. 1992) and Taiwan (Wang 1958), these records have been noted as misidentifications or are considered doubtful (Shear 1999; Mikhaljova and Lim 2000, 2008; Mikhaljova and Korsós 2015). Lim (2001) also recorded *D. gracilipes* from Jeju Island, Korea, but provided no specific photographs or illustrations. There are also records from “Honshu”, Japan (Takakuwa and Takashima 1944; Takashima 1949; Takakuwa 1954), but these lack specific locality data. Furthermore, Ishii (1991, 2004) recorded this species from the Kanto region of Honshu, but no photographs or illustrations were provided, and the basis for identification was not stated. Since the family Diplomaragnidae is classified based on gonopod morphology (Shear 1990; Mikhaljova 2000), records lacking identification characters are treated as doubtful in the present study. Consequently, reliable records of *D. gracilipes* are currently known only from Hokkaido.

## Discussion

**Figure 9.**
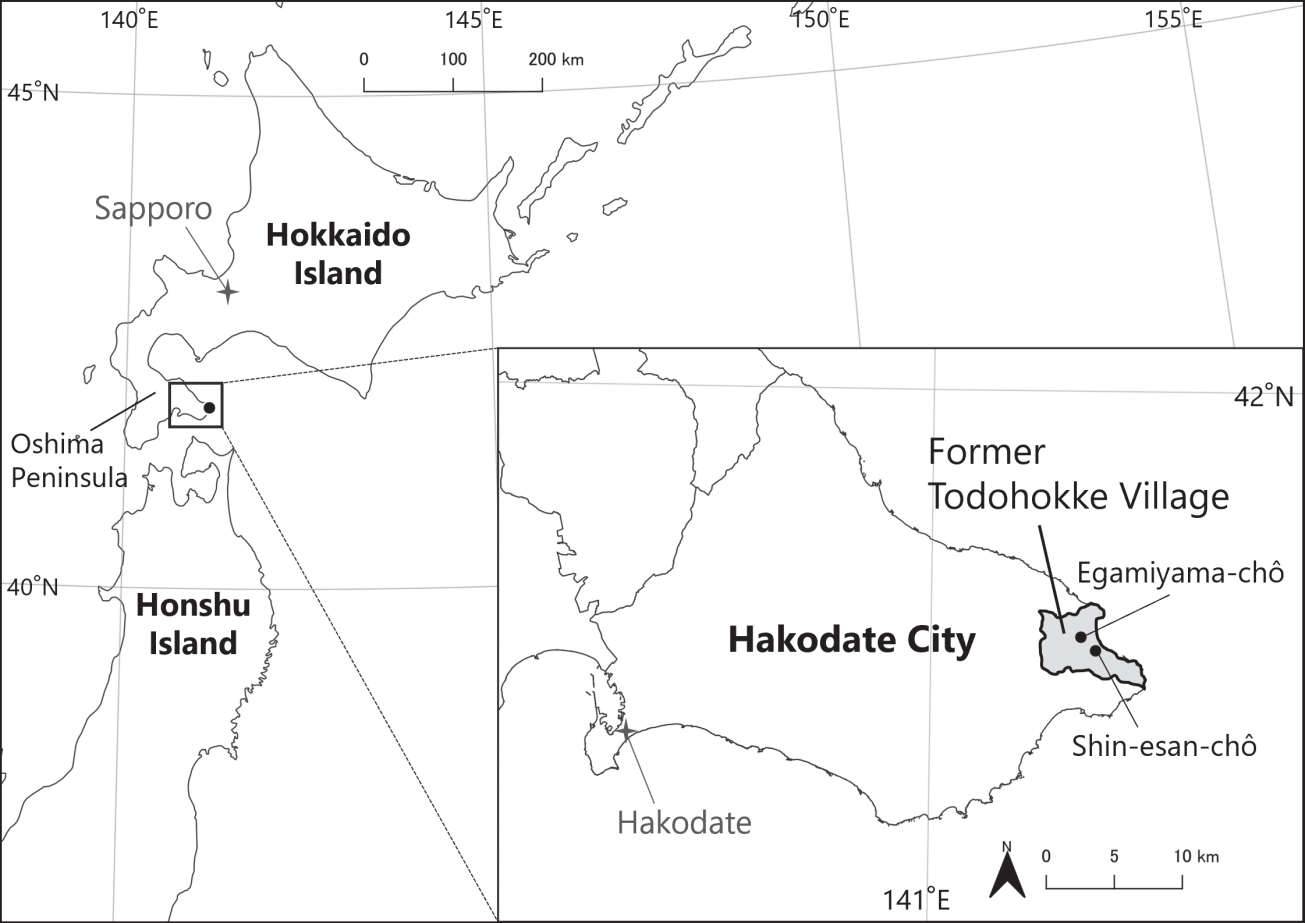
Collection site. Closed circles, locality of specimens examined.

Attems (1927) described *Escaryus japonicus* Attems, 1927, based on specimens collected by Mr. Sauter in Japan. The type locality of *E. japonicus* was described as “Todohokhe, Hohango, Japan” (Attems 1927). Since no such Japanese place names as “Todohokhe” or “Hohango” exist, it has been pointed out that it is difficult to determine the type locality of *E. japonicus* (Murakami 1972). On the other hand, Ilie et al. (2009) registered the type series of Geophilomorpha preserved in the Natural History Museum in Vienna and listed the type locality of *E. japonicus* as “Japan, Todohokke, Hokaido?”. Although the collection date was not provided in Attems (1927), Ilie et al. (2009) clarified that the collection date was “1 November 1904”. “Todohokke” is considered to correspond to the former Todohokke Village (椴法華村), located in the eastern part of the Oshima Peninsula, Hokkaido, Japan (Fig. 9). This village existed from 1876 to 2004 (Hakodate City 2022) and is now incorporated into Hakodate City, Hokkaido, Japan (Fig. 9).

A review of Sauter’s collection records from Hokkaido, Japan (Table 2), confirmed that, besides *E. japonicus* (Attems 1927; Ilie et al. 2009), *Enteroctopus dofleini* Wülker, 1910 (Octopoda: Enteroctopodidae) and *Ommastrephes sagittatus* (Lamarck, 1798) (Oegopsida: Ommastrephidae) were also collected in Todohokke (Wülker 1910). Furthermore, *Zanclea* sp. (Anthoathecata: Zancleidae) was collected on 2 November 1904, although a detailed locality was not provided (Maas 1909). Mr. Sauter is thought to have stayed in Japan from 1902 to 1906 (Pannhorst 2016a, 2016b; Yamauchi et al. 2026), and his collection records suggest that he likely visited Hokkaido only in November 1904 (Yamauchi et al. 2026).

**Table 2.** Mr. Hans Sauter’s collection records from Japan.

**Collection site (original spelling)**	**Collection date**	**Species collected**	**References**
Hokkaido	2-Nov-04	*Zanclea* sp.	Maas (1909)
Todohokke (Hokkeido)	Unknown	*Enteroctopus dofleini* Wülker, 1910	Wülker (1910)
		*Ommastrephes sagittatus* (Lamarck, 1798)	
Hokkaido	Unknown	*Japanosoma scabrum* Verhoeff, 1914	Verhoeff (1914)
		*Macrochaeteuma sauteri* Verhoeff, 1914	
		*Diplomaragna gracilipes* (Verhoeff, 1914)	
Todohokhe, Hohango	Unknown	*Escaryus japonicus* Attems, 1927	Attems (1927)
Todohokke, Hokaido?	1-Nov-04		Ilie et al. (2009)

Based on the above, it is presumed that Mr. Sauter also collected millipedes in the area of the former Todohokke Village, Hokkaido, in November 1904. *Japanosoma scabrum* and *M. sauteri* were collected in what is now Shin-esan-chô, Hakodate City, and *D. gracilipes* was collected in what is now Egamiyama-chô, Hakodate City; both localities fall within the boundaries of the former Todohokke Village (Fig. 9). The specimens of *J. scabrum*, *M. sauteri*, and *D. gracilipes* collected in the present study are consistent with the characteristics of the original descriptions by Verhoeff (1914). Therefore, it is highly probable that their type locality is the former Todohokke Village, Hokkaido, Japan. If Mr. Sauter indeed collected these species in November 1904, the records presented here represent the first reliable records in approximately 120 years.

## Supplementary Material

XML Treatment for Japanosoma scabrum

XML Treatment for Macrochaeteuma sauteri

XML Treatment for Diplomaragna gracilipes

## Additional information

### Artificial Intelligence (AI) use

Regarding the use of AI in the preparation of this manuscript, the authors declare the following:

AI-assisted technology was used to edit language and check English expressions for clarity.

### Author ORCIDs

N. Hirakizawa https://orcid.org/0009-0005-1745-1160

R. Kuwahara https://orcid.org/0009-0006-8889-0205

T. Yamauchi https://orcid.org/0009-0006-8179-8254

### Data availability

All of the data that support the findings of this study are available in the main text.
